# Supplementary material for: Global hypo-methylation in a proportion of glioblastoma enriched for an astrocytic signature is associated with increased invasion and altered immune landscape
Source: eLife. 2022 Nov 22;11:e77335. doi: 10.7554/eLife.77335 (PMC9681209; doi:10.7554/eLife.77335)
Supplement: Figure 2—source data 1. [file elife-77335-fig2-data1.zip › Figure_2_source_data_1/Figure_2I_J/homerResults/motif15.similar.html]

motif15

## Information for motif15

C
T
A
G
A
T
C
G
C
G
A
T
G
C
A
T
C
G
T
A
C
T
A
G
A
C
T
G
C
A
G
T
  
Reverse Opposite:  

G
T
C
A
A
G
T
C
A
G
T
C
G
C
A
T
C
G
T
A
C
G
T
A
A
T
G
C
A
G
T
C
  

|  |  |
| --- | --- |
| p-value: | 1e-39 |
| log p-value: | -9.033e+01 |
| Information Content per bp: | 1.838 |
| Number of Target Sequences with motif | 45.0 |
| Percentage of Target Sequences with motif | 6.20% |
| Number of Background Sequences with motif | 0.7 |
| Percentage of Background Sequences with motif | 0.26% |
| Average Position of motif in Targets | 108.3 +/- 52.3bp |
| Average Position of motif in Background | 109.0 +/- 0.0bp |
| Strand Bias (log2 ratio + to - strand density) | 0.2 |
| Multiplicity (# of sites on avg that occur together) | 1.07 |
| Motif File: | file (matrix) reverse opposite |

### Similar de novo motifs found

|  |  |  |  |  |  |  |  |
| --- | --- | --- | --- | --- | --- | --- | --- |
| Rank | Match Score | Redundant Motif | P-value | log P-value | % of Targets | % of Background | Motif file |
| 1 | 0.784 | A C T G A C T G A C G T A C G T G T C A A C T G | 1e-7 | -17.661250 | 5.10% | 2.03% | motif file (matrix) |
